# Prevalence and correlates of depression among Nigerian stroke survivors

**DOI:** 10.4102/sajpsychiatry.v25i0.1252

**Published:** 2019-05-20

**Authors:** Olushola Olibamoyo, Abiodun Adewuya, Bolanle Ola, Olurotimi Coker, Olayinka Atilola

**Affiliations:** 1 Department of Psychiatry, Lagos State University Teaching Hospital, Lagos, Nigeria; 2 Department of Behavioural Medicine, Lagos State University College of Medicine, Lagos, Nigeria

**Keywords:** Post-Stroke depression, Lesion location, Stroke severity, Disability, Socio-demographic, Clinical factors

## Abstract

**Background:**

There is mixed evidence for the hypothesis that the risk of depression after stroke is influenced by the location of lesions in the hemispheres, demographic and clinical factors, and disability of stroke survivors.

**Aim:**

The current study determined the prevalence of depression and its socio-demographic and clinico-pathological correlates among stroke survivors in a tertiary hospital in Lagos, Nigeria.

**Method:**

The cross-sectional study was carried out among 112 adult patients with a clinical history of stroke confirmed by neuroimaging. Depression was diagnosed using Mini International Neuropsychiatric Interview. The socio-demographic profile was obtained, and cognitive impairment was assessed using the Mini-Mental State Examination. Stroke severity was assessed retrospectively using the National Institute of Health Stroke Scale and current disability was measured using the Modified Rankin Scale.

**Results:**

There were 48 (42.9%) stroke survivors with a clinical diagnosis of depression. Using binary logistic regression, the independent determinants of depression were younger age, unemployment, perceived poor social support, increasing number of previous admissions because of stroke, cognitive impairment, severity of stroke and current disability status. However, there was no significant association between depression and lesion location.

**Conclusion:**

Depression is a common associate of stroke, and there is a need for sustained focus on young stroke survivors with severe stroke, especially those who do not have social support and have low socio-economic status, who may have a higher risk of developing depression following stroke.

## Introduction

Stroke is a recognised medical emergency,^1^ and it has remained a major health problem worldwide because of its high prevalence and because it is a leading cause of adult physical disability.^2^ Globally, about 10.3 million new cases of stroke yearly and 25.7 million stroke survivors were estimated in 2013,^3^ and it was among the top 10 leading causes of years of life lost worldwide in 2016.^4^ Stroke is defined as rapidly developing clinical signs of focal or global disturbance of cerebral functions, with symptoms lasting 24 h or longer or leading to death with no apparent cause other than that of vascular origin.^5^

The World Health Organization estimates suggest that 8% of all first-ever strokes occur in Africa and that 5% of stroke survivors worldwide live in Africa^5^; further, the burden of stroke in low- and middle-income countries accounts for over 87% of disability adjusted life years.^6^ This is a result of overwhelming costs incurred by the health systems and complications being under-recognised and undertreated.^7^

A stroke survivor is an individual who has been diagnosed as having a stroke, regardless of the level of handicap.^8^ Among survivors, neuropsychiatric disturbances are frequently seen in clinical practice, and the commonest is depression.^8^ Prevalence of post-stroke depression (PSD) from studies worldwide varies from 9% to 53%,^9,10,11^ while Nigerian studies of PSD also have rates varying from 16.2% to 52.9%.^12,13,14,15,16,17,18,19,20,21,22^ Post-stroke depression has profound effects on the lives of both survivors and caregivers.^23^

Two theories of PSD have been largely adopted. The first suggests that PSD is a result of specific brain lesion and presumably subsequent changes in neurotransmitters, while the second describes it as a psychological reaction to the clinical consequence of stroke. Previous studies on PSD and lesion location showed that the association is influenced by patient’s selection, study design and time difference of measurement of depression.^10^

Moreover, earlier reviews mostly found stroke-related factors (lesion location, cognitive impairment and severity of stroke) to be responsible for depression to a large extent; however, in addition to these, different patient-related factors are found equally responsible in causing PSD, for example, age, gender, educational level and social support.^24^

Severity of impairment in activities of daily living and functional status has been frequently associated with PSD. Most studies on PSD found a statistically significant relation between the severity and existence of PSD and severity of impairment of activities of daily living.^25,26^

This study will provide information about PSD in Nigeria and simultaneously evaluate the relationship between PSD and the socio-demographic and clinico-pathological correlates of depression among survivors. Such an approach aids better understanding of PSD, given that depression in the general population is multi-causal.

## Method and materials

### Setting

This was a cross-sectional study that was carried out at the neurology outpatient department of the Lagos State University Teaching Hospital, Ikeja. The clinic receives an average of 150 stroke-related follow-up patients in a year.

### Participants

The respondents were adult stroke survivors (aged 18–65 years). The inclusion criteria were a previous history of stroke not less than 6 months prior and confirmation by neuroimaging (computed tomography scan–magnetic resonance imaging) of the brain within the acute episode of stroke (within 7 days of stroke).

Exclusion criteria were history of any psychotic disorder, history of pre-stroke mood disorder, language impairment severe enough to prevent neuro-psychiatry assessment, history of other central nervous disorders (head injury, multiple sclerosis, etc.) and acute illness preventing proper assessment.

A calculated sample size of 112, from a finite population of 150 regular follow-up patients, was needed for a 95% confidence interval in detecting a margin of error of 0.05 using a prevalence of 30.0% from a similar study^15^ as prior judgement of the correct value and 10% attrition rate.

### Instruments

A pro forma questionnaire was used to collect socio-demographic data, which included a question on the level of social support received from family and friends, while clinical variables about stroke location (neuroimaging report), stroke type, stroke severity and number of stroke episodes were obtained from participants’ case files:

1. Depression was assessed using the depression module of the Mini International Neuropsychiatric Interview (MINI) English Version 5.0.0. The MINI has an acceptably high validation and reliability score and can be administered in a much shorter period of time when compared to the Structured Clinical Interview for DSMIII, Patient edition and Composite International Diagnostic Interview. It has been used in various studies in Nigeria.^27^
2. Mini-Mental State Examination (MMSE): The MMSE is an interviewer-rated instrument used in assessing cognitive function. The maximum score is 30, with normal individuals scoring 24 and above, while a score of less than 24 indicative abnormal cognitive function. It has been used in Nigerian studies.^28^
3. National Institute of Health Stroke Scale (NIHSS): The NIHSS is an objective clinical tool used in the evaluation of stroke severity. It comprises 15 items of neurologic examination. It is scored from 0 to a maximum of 42. A high NIHSS score signifies severe stroke, and it has been shown to be the best predictor of outcome. It has been validated in Nigeria; a score equal to or greater than 16 forecasts a high probability of severe disability and a score of less than 16 forecasts good recovery.^29^ The admission stroke severity was obtained from the case files retrospectively using NIHSS.
4. Modified Rankin Scale (MRS): The MRS measures independence rather than performance of specific tasks. The scale consists of six grades from 0 to 5. Patients with a score of 0–3 were categorised as having a good outcome (not handicapped) and those with a score of 4–5 as handicapped.^30^ It has been used in Nigerian studies of stroke survivors.^17^

### Procedure

Participants were recruited using simple random sampling without replacement, where seven participants were picked from an average of 10 stroke survivors each clinic day via balloting. Recruitment was carried out over 8 weeks. None of the recruited participants declined consent.

Literate patients were allowed to complete the pro forma questionnaire on their own, while those who were not had their answers recorded accordingly. The MINI, MRS, NIHSS and MMSE were administered by researchers who were trained on the use of the MINI prior to data collection, during which inter-rater reliability was measured. The diagnosis of stroke was made by a consultant neurologist, while neuroimaging was reported by a consultant radiologist. The participants who had depression were informed about it, and their managing physicians were also informed about the need to treat.

### Data analysis

The data were coded, entered into a statistical package and cleaned. The statistical analysis was performed using SPSS version 23. Categorical variables were summarised with frequencies and percentages, while continuous variables were summarised with their mean, median, range and standard deviation.

The sampled population was divided into diagnostic groups of depressed and non-depressed individuals. The chi-square test was used to find the significance of study parameters on categorical variables, while the Mann–Whitney U test was used to analyse continuous variables (they were not normally distributed) between the two groups. The *p*-value was set at 0.05. Independent predictors of depression were determined by binary logistic regression. A *p*-value of less than 0.05 was considered significant.

## Ethical consideration

Approval (LREC/06/10/732) was obtained from the Ethics and Research Committee of Lagos State University Teaching Hospital (NHREC04/04/2008) before commencement of the study. Written informed consent was obtained from all participants after the aims, objectives and purpose of the study were explained to them. Confidentiality and voluntariness regarding participation were also explained.

## Results

A total of 112 patients were recruited, assessed and analysed. The same number was analysed. The mean age was 56.71 ± 6.49 years, and there were 67 (59.8%) males. Eighty (71.4%) were married, 71 (63.4%) were from the Yoruba ethnic group and 78 (69.6%) were Christians.

Up to 47 (42.0%) had tertiary education, while 67 (59.8%) were self-employed. The majority (66; 58.9%) collected a monthly salary or allowance between $70 and $130, while 85 (75.9%) were living with their nuclear family. Moreover, 90 (80.4%) rated their perceived social support from family members as fair.

Neuroimaging of both MRI and CT scans showed that 62 (55.4%) had right hemisphere stroke, while 88 (78.6%) experienced infarction. The mean duration of stroke was 51.78 ± 46.21 months. The majority of respondents (94; 83.9%) had had only one episode of stroke, whereas five (4.5%) had had three episodes. Only seven (6.3%) respondents had had three or more hospitalisations resulting from stroke. The mean MMSE score was 27.42 ± 2.42, and eight (7.1%) respondents had mild cognitive impairment.

The mean NIHSS score was 15.46 ± 3.00, and 47 (42.0%) participants scored 16 and above, which forecasted severe stroke. The mean MRS score was 1.69 ± 1.19, and 12 respondents (10.7%) showed no symptoms.

The rate of major depressive disorder using the MINI was found to be 42.9% in the studied population.

### Correlates of depression among stroke survivors

Post-stroke depression was significantly (*U* = 967.50, *p* = 0.001) associated with younger age, as indicated in the Mann–Whitney U test; hence, depression in stroke survivors decreases with increasing age.

There is a significant risk (*p* < 0.001) of PSD in survivors who are unemployed (78.6%) compared to those employed by others (35.5%); however, being self-employed carried the least risk of having depression (29.9%). Further, Mann–Whitney *U* test indicated that PSD was significantly (*U* = 1051.50, *p* = 0.004) associated with lower monthly allowance.

Survivors with good social support had 16.7% risk of depression compared with 90.0% by survivors with poor social support, which was statistically significant (*p* = 0.001). Moreover, 34.7% of stroke survivors with one previous admission had PSD; this rate increased to 80.0% in survivors with two previous admissions and practically 100% in survivors with three or more previous admissions. For cognitive function, 38.5% of those without cognitive impairment had PSD, while 100% of those with mild cognitive impairment had PSD.

There were 13 patients scoring 4–5 on the MRS, and 12 (92.3%) of them had PSD, compared to 36.4% of patients with MRS scores between 0 and 3 who had PSD (*p* < 0.001). In the same vein, out of the 47 who scored 16 and above on the NIHSS, 40 (85.1%) participants were depressed compared to 12.3% of those who scored 0–15 who had PSD. This result was significant (*p* < 0.001).

Localisation of stroke characteristics, which was taken from CT and MRI reports of survivors, revealed a higher level of depression in the left hemisphere (52.1%) compared to the right hemisphere (35.5%); however, PSD and stroke localisation had no statistically significant association (*p* = 0.214). The rest of the results are summarised in Table 1 to Table 3

**TABLE 1:** Test of association between depression and socio-demographic characteristics using chi-square.

Variables	Total	Depressed (*n* = 48)		Non-depressed (*n* = 64)		Test of significance
	(*n* = 112)	*n*	%	*n*	%	
Gender						
Females	45	23	51.10	22	48.90	χ^2^ = 2.093
Males	67	25	37.30	42	62.70	*df* = 1
						*p* = 0.148
Marital status						
Presently not married	32	14	43.80	18	56.20	χ^2^ = 0.150
Presently married	80	34	42.50	46	57.50	*df* = 1
						*p* = 0.904
Ethnicity						
Yoruba	71	27	38.00	44	62.00	χ^2^ = 1.847
Other	41	21	51.20	20	48.80	*df* = 1
						*p* = 0.174
Religion						
Christianity	78	33	42.30	45	57.70	χ^2^ = 0.032
Islam	34	15	44.10	19	55.90	*df* = 1
						*p* = 0.859
Level of education						
Primary or less	16	9	56.20	7	43.80	χ^2^ = 1.416
Secondary	43	18	41.90	25	58.10	*df* = 2
Tertiary and above	53	21	39.60	32	60.40	*p* = 0.493
Employment status						
Unemployed	28	22	78.60	6	21.40	χ^2^ = 19.609
Employed	17	6	35.50	11	64.50	*df* = 2
Self-employed	67	20	29.00	47	71.00	*p* < 0.001*
Perceived social support						
Good	12	2	16.70	10	83.30	χ^2^ = 13.749
Fair	90	37	41.10	53	58.90	*df* = 2
Poor	10	9	90.00	1	10.00	*p* = 0.001*

χ^2^ = chi-square; *df*, degree of freedom.

*p* = level of significance

* < 0.05; bold numbers indicate statistical significance.

**TABLE 2:** Test of association between depression and clinical or illness-related characteristics using chi-square.

Variables	Total (*n* = 112)	Depressed (*n* = 48)		Non-depressed (*n* = 64)		Test of significance
		*n*	%	*n*	%	
**Previous admission**						
1	95	33	34.7	62	65.3	**χ^2^ = 17.525**
2	10	**8**	**80**	2	20	***df* = 2**
3 or more	7	**7**	**100**	0	0	*p* < 0.001*
**Neuroimaging**						
Left hemisphere	48	25	52.1	23	47.9	χ^2^ = 3.086
Right hemisphere	62	22	35.5	40	64.5	*df* = 2
Both hemispheres	2	1	50	1	50	*p* = 0.214
**Aetiopathogenesis**						
Infarction	88	38	43.2	50	56.8	χ^2^ = 0.018
Haemorrhage and thrombotic	24	10	41.7	14	58.3	*df* = 1
						*p* = 0.894
**MMSE**						
24–30 (no impairment)	104	40	38.5	64	61.5	**χ^2^ = 9.112**
18–23 (mild impairment)	8	**8**	**100**	0	0	***df* = 1**
						*p* = 0.003*
**MRS**						
0–3 (good outcome and not handicapped)	99	36	36.4	63	63.6	**χ^2^ = 59.028**
4–5 (handicapped)	13	12	92.3	1	7.7	***df* = 1**
						*p* < 0.001*
**NIHSS**						
0–15 (forecast good recovery)	65	9	12.3	57	87.7	**χ^2^ = 12.490**
16 and above (forecast severe stroke)	47	40	85.1	7	14.9	***df* = 1**
						*p* < 0.001*

χ^2^ = chi-square; *df*, degree of freedom; MMSE, Mini-Mental State Examination; MRS, Modified Rankin Scale; NIHSS, National Institute Health Stroke Scale.

*p* = level of significance

* < 0.05; bold values indicate statistical significance.

**TABLE 3:** Test of association between depression and clinical and socio-demographic characteristics using the Mann–Whitney U test.

Variables	Mean rank	Sum of ranks	Mann–Whitney *U*	Z	*p*
**Age (years)**					
Depressed	44.66	2143.50	**967.50**	**−3.352**	**0.001**
Not depressed	65.38	4184.50			
**Monthly allowance (dollars)**					
Depressed	46.41	2227.50	**1051.50**	**−12.872**	**0.004**
Not depressed	64.07	4199.50			
**Illness duration (months)**					
Depressed	49.84	2392.50	1216.50	−1.903	0.057
Not depressed	61.49	3935.50			
**MMSE**					
Depressed	32.55	1562.50	**386.50**	**−6.869**	**< 0.001**
Not depressed	74.46	4765.50			
**MRS**					
Depressed	80.69	3873.00	375.00	−7.204	**< 0.001**
Not depressed	38.36	2455.00			
**NIHSS**					
Depressed	83.79	4022.00	226.00	−8.329	**< 0.001**
Not depressed	36.03	2306.00			

MMSE, Mini-Mental State Examination; MRS, Modified Rankin Scale; NIHSS, National Institute Health Stroke Scale.

*p* = level of significance < 0.05; bold values indicate statistical significance.

### Independent determinants of post-stroke depression

The independent determinants of PSD analysed by binary logistic regression are shown in Table 4. Determinants include age (*p* = 0.001, adjusted odds ratio [OR] 0.030, 95% confidence interval [CI] = 0.01–0.220), stroke severity (*p* < 0.001, OR = 49.062, CI = 9.593–250.925), previous admission (*p* < 0.001, OR = 53.515, CI = 6.633–431.776), disability status (*p* < 0.001, OR = 4.234, CI = 2.456–7.231), as well as employment status, cognitive impairment and perceived social support.

**TABLE 4:** Binary logistic regression showing independent predictors of depression among 112 Nigerian stroke survivors.

Variables	Adjusted odds ratio	95% confidence interval	*p*
**Age group (years)**			
< 50	1		
50–60	0.122	0.120–0.666	**0.015**
> 60	0.030	0.010–0.220	**0.001**
**Employment status**			
Unemployed	1		
Employed	0.094	0.009–0.925	**0.043**
Self-employed	0.105	0.027–0.412	**0.001**
**Monthly allowance (dollars)**			
0–130	1		
> 130	1.043	0.027–0.412	0.959
**Social support**			
Good	1		
Fair	7.633	0.957–59.614	0.056
Poor	92.431	3.712–2296.83	**0.006**
**Previous admission**			
1	1		
≥ 2	53.515	6.633–431.776	**< 0.001**
**NIHSS**			
0–15	1		
≥ 16	49.062	9.593–250.925	**< 0.001**
**MRS**			
0–3	1		
4–5	4.234	2.456–7.231	**< 0.001**
**MMSE**			
24–30	1		
18–23	15.000	5.826–38.660	**< 0.001**

MMSE, Mini-Mental State Examination; MRS, Modified Rankin Scale; NIHSS, National Institute Health Stroke Scale.

*p* = level of significance < 0.05; bold values indicate statistical significance.

## Discussion

The main findings of the present study were prevalence of PSD of 42.9% and that the independent determinants of PSD were decreasing age, unemployment, perceived poor social support, increasing number of previous hospital admissions because of stroke, stroke severity, present disability status and cognitive impairment.

This prevalence is consistent with that witnessed in similar studies in Nigeria, which reported a range from 40% to 42%;^16,19,20^ however, it is much higher than the 22.9%^22^ and 25.5%^12^ found in Lagos and lower than the 52.9% reported by Mshelia et al.,^21^ also conducted in Nigeria. It is to be noted that certain other studies conducted locally have found prevalences ranging from 16.2% to 30%.^13,14,15,17,18^

Overall, the 42.9% found in the present study is consistent with hospital-based studies of PSD in other parts of the world, which reported a rate ranging from 35% to 53%,^31,32^ whereas community-based studies reported lower rates, ranging from 9% to 23%.^11,33^

A close observation of the possible reasons for this variability may be connected with (1) the number of patients assessed and (2) methodological problems in recognising, assessing and diagnosing depression. Local studies with sample sizes of less than 100 patients for PSD reported a prevalence of 30% or less. It is possible that these studies either did not consider sample sizes that were representative of the base populations or used small samples that were not sufficient to give the studies the required power to address the hypothesis, which may be responsible for the lower prevalence reported.

Moreover, most of the studies that used diagnostic tools reported higher prevalence compared to those that used screening instruments. This difference may be explained by the presence of physical symptoms like poor sleep and fatigue seen during the post-stroke period, which are also part of the diagnostic assessment of depression and may cause over-diagnosis; however, in this study, participants were assessed 6 months post-stroke to reduce this possibility.

For age, the result is in line with the study conducted by Mshelia et al.^21^ which found 73.8% of participants aged less than 35 years were depressed and the differences were statistically significant (*p* = 0.002). This is in tandem with various studies^34,35^ that noted that PSD was more prevalent in the younger age group. Conversely, older age has been found to be significantly predictive of developing PSD in a number of studies.^17,31^ However, numerous studies have failed to yield a significant difference between age groups and PSD.^15,36^

This may be because survivors usually experience a reduced sense of control and a shift in life perspective, as stroke is recognised as taking away an anticipated future^37^; further, the onset of such an acute physical health problem requires an individual to adapt rapidly in a highly emotional and changeable situation. Older people have a tendency to accept their difficulties because of past life experiences, resilience, declining optimism and physical ability, which provide a possible realistic adjustment and coping mechanisms and may be a protective factor.

Other emotional coping mechanisms such as having a health-focussed network of support and being familiar with healthcare systems are usually more readily available to older individuals compared to the younger ones, who feel despondent with a sudden and unexpected cessation of their dreams and aspirations following stroke and may feel unjustly deprived of a healthy life.

Regarding social support, the poorer it is, the higher the risk of PSD. This is consistent with other studies conducted locally^38,39^ and internationally.^40^ According to both the stress process model^41^ and the transactional model of stress and coping,^42^ the outcome of a stressful event depends highly on the coping resources available. It is plausible in this case that social support serves as one of the effective coping mechanisms among stroke survivors. This may in turn influence their mental health. This explains why stroke survivors with good social support reported less depression.

It has been shown that the perception of availability of social support has the potency to make a person perceive a stressful situation as less stressful.^43^ This enhances the individual’s capacity to cope with the situation. However, once depressive symptoms set in, the influence of social support becomes less significant; this may be because of a depressed stroke survivor having a reduced tendency to perceive the availability of social support, hence reducing the reality and perception of social support.

The result of PSD and cognitive impairment is consistent with that found in a similar study in Nigeria showing cognitive dysfunction as measured by MMSE to be significantly associated with PSD^16^; however, it is noteworthy that another study conducted in Nigeria found no statistically significant association between PSD and cognitive impairment.^19^

However, most studies have reported that stroke patients with major depression have a greater degree of cognitive impairment than non-depressed patients.^35^ This association could be explained by either cognitive impairment causing depression, as suggested by Anderson et al.,^44^ or that depression leads to a greater degree of cognitive impairment, as corroborated by the fact that serotonin receptors binding in several brain regions correlated significantly with the MMSE scores of patients with PSD and cognitive impairment.^45^ Furthermore, patients who were effectively treated for PSD showed sustained improvement of cognitive functions over a 2-year period.^46^

Many studies have examined the issue of whether persons with severe stroke are more likely to develop PSD, and among them are studies conducted in Nigeria^12,18^ that found a statistically significant association between the severity of stroke and a greater risk of PSD.

Post-stroke depression is associated with functional ability and may have a negative impact on recovery. The result is consistent with findings from other similar studies, which reported that impaired functional status and disability measured by MRS or any other disability measure is significantly associated with PSD.^12,21,47,48^ This may be a result of the fact that after surviving a stroke, the majority of patients must cope with a multitude of disabilities in physical, psychological and social functioning. This burden of stroke can increase the risk of PSD, which then leads to further impairment like increased disability, reduced social activities, delayed recovery, failure to return to work and longer institutional care.

Conversely, depression in patients with chronic conditions like stroke is associated with poor outcomes, thereby sustaining severe levels of disability or worsening it. This is plausible through a number of bio-behavioural mechanisms like lack of treatment adherence, as well as lifestyle factors such as smoking and physical inactivity. Moreover, effective management of PSD improved not only patients’ mood but their functionality as well.^49^

Post-stroke depression was not significantly associated with the hemispheric side of the lesion location for the study. The result of this study is in line with other local studies that looked at the association between lesion location and depression among stroke survivors.^21,22^ No consensus can be found in the literature about this association, which remains controversial.

This study has some limitations, which include the cross-sectional nature of the study, which has inherent limitations for interpretation of not allowing inferences of causality to be made. One tertiary study setting limits its generalisability in the community setting. Furthermore, the exclusion of patients with aphasia means that the results cannot be generalised to those stroke patients who have aphasia. It is equally important to note that social support was assessed in the study based on the perception of stroke survivors, notwithstanding the actual availability of support. This should be taken into consideration in interpreting findings of social support with PSD, although it is believed that social support is a subjective construct that centres primarily on the patient’s perception and that its assessment should be based on perception.^50^

However, this is one of the few studies from Nigeria that made use of neuroimaging to make an exact definitive diagnosis, thus avoiding the possibility of misdiagnosis and inclusion of other neurological diagnoses with similar clinical presentation as stroke.

The study also used a diagnostic tool, the MINI, which can be used in primary care where the patients present, hence its applicability at routine clinics.

In conclusion, the PSD prevalence of 42.9% suggests that clinicians have to acknowledge that depression is a common outcome after stroke, especially when none of the stroke survivors who were studied had previous psychiatric assessment for depression. Furthermore, special attention should be given to stroke survivors with cognitive impairment who are younger (< 50 years), have poor social support, previous hospitalisation, low socio-economic status, more severe stroke and higher disability.
